# A Comparison of Static and Shake-Flask Cultures for Filamentous Fungal Biomass and L-Carnitine Production in Rum Stillage

**DOI:** 10.3390/jof12090675

**Published:** 2026-09-09

**Authors:** Hashini J. Wahalathanthrige, Aline D. O. Campos, Zhanying Zhang, P. James Strong

**Affiliations:** 1 Centre for Agriculture and the Bioeconomy, Queensland University of Technology, Brisbane 4000, Australia; 2 School of Biology and Environmental Science, Faculty of Science, Queensland University of Technology, Brisbane 4000, Australia; 3 School of Mechanical, Medical and Process Engineering, Faculty of Engineering, Queensland University of Technology, Brisbane 4000, Australia; 4 New Zealand Institute for Bioeconomy Science Limited, Te Papa Tipu Innovation Park, 11 Titokorangi Drive, Rotorua 3010, New Zealand

**Keywords:** vinasse, dunder, mycoprotein

## Abstract

This study investigated the potential of Bundaberg rum stillage as a low-cost medium for cultivating two food-related filamentous fungi: *Aspergillus oryzae* and *Rhizopus oligosporus*. Traditional shake-flasks and a novel static-liquid culture fermentation approach were used to assess biomass and L-carnitine synthesis. Stillage was assessed to determine the optimal concentration for use, and this was applied throughout. Growth was benchmarked against a complex synthetic medium containing glucose, yeast extract and malt extract. The highest biomass yields were obtained in the stillage supplemented with yeast extract, *Aspergillus oryzae* static culture (5.84 g/L), while *R. oligosporus* favoured agitated culture (4.82 g/L). *Aspergillus oryzae* produced the highest yield of L-carnitine (3.71 mg/g) in the stillage supplemented with ammonium in an agitated culture, while *R. oligosporus* synthesised 3.39 mg/g of L-carnitine under an agitated culture in the unsupplemented stillage. Volumetric yields of L-carnitine underscored the importance of aeration and nitrogen availability for metabolite synthesis, where both *A. oryzae* (15.8 mg/L) and *R. oligosporus* (12.7 mg/L) performed best in agitated ammonium-supplemented stillage. These findings demonstrate that the optimal cultivation strategy is product-specific, with static cultivation and yeast extract supplementation favouring fungal biomass production, while better aeration and an ammonium supplement improved L-carnitine yield. The results provide a basis for developing tailored bioprocesses for the commercial valorisation of rum stillage, enabling the sustainable production of fungal biomass and value-added metabolites while supporting circular bioeconomy approaches for distillery wastewater management.

## 1. Introduction

There is a global trend towards food that serves a function beyond the traditional provision of protein and energy. Functional foods enriched with bioactive and beneficial compounds are becoming increasingly sought after. Broad groups of compounds, such as vitamins, polyphenols, and antioxidants, have demonstrated therapeutic effects that support human health and well-being [1]. Some filamentous fungi produce mushrooms, which have served as a dietary source of protein, lipids, fatty acids, vitamins, and fibre for millennia. Mushrooms have long been recognised for their bioactive constituents such as β-glucans and triterpenoids. Besides mushrooms, fungal mycelia are also utilised as a primary product and frequently serve as key ingredients in nutritional or therapeutic supplements due to their diverse specialised metabolites [2].

Previous studies using *Pleurotus*, *Agaricus*, *Ganoderma*, *Trametes*, *Aspergillus* or *Rhizopus* species in stillage have demonstrated their ability to produce health-benefiting compounds such as chitosan, L-carnitine, triterpenoids and β-glucans, while simultaneously reducing the soluble organic load [3,4,5,6,7]. Of these bioactive compounds, L-carnitine is a quaternary ammonium compound that facilitates fatty acid oxidation in human metabolism [8]. This process is crucial for non-oxidative glucose metabolism in the human body, and particularly important for patients with type 2 diabetes. Furthermore, L-carnitine plays a vital role in muscle function during intensive exercise, cardiovascular health, weight management and cognitive performance [9]. L-carnitine is obtained either through endogenous biosynthesis or dietary supplementation [10]. Out of the total L-carnitine intake, 75% is derived from animal-based foods such as meat and dairy products, whereas plant-based sources are limited [11]. Additionally, L-carnitine can be chemically synthesised using precursors such as epichlorohydrin and trimethylamine [12]. However, the synthesis of enantiomerically pure L-carnitine requires chiral resolution or stereospecific synthesis processes that are often environmentally hazardous [13,14]. Advances in biotechnological approaches have therefore focused on microbial-based production systems that provide more cost-effective and environmentally sustainable alternatives [11].

Filamentous fungi are promising candidates that are capable of fermentative L-carnitine biosynthesis, offering an efficient and sustainable alternative to animal-derived sources for L-carnitine-rich foods [7,15]. Previous studies have demonstrated L-carnitine production using *Aspergillus* and *Rhizopus* species in solid-state fermentation with buckwheat, wild ginseng, and quinoa [16,17,18]. Although solid-state fermentation is not energy intensive, scale-up is still challenging, and the final product often contains unutilised substrate. Consequently, the submerged cultivation of GRAS (Generally Recognised as Safe) filamentous fungi, capable of producing L-carnitine, offers a more efficient alternative, as the fungal biomass can be easily recovered using a low-energy recovery method such as crude filtration. Among strains tested in a semi-synthetic medium, *Aspergillus oryzae* exhibited the highest potential for L-carnitine synthesis under submerged conditions relative to *Rhizopus oryzae*, *Rhizopus oligosporus* and *Neurospora intermedia* [7].

In liquid bioprocesses, static-liquid culture and culture fundamentally differ in mass transfer efficiency, morphological development, and overall process economics [19]. Shake-flask and stirred-tank fermenters provide continuous mechanical agitation, which strongly improves oxygen transfer rates (kLa) and ensures homogeneous nutrient distribution throughout the bulk liquid [19,20]. The relatively high oxygen availability suppresses anaerobic fermentative pathways, accelerates vegetative growth, and often promotes dense hyphal pelleting or dispersed mycelial branching [19,21]. However, intensive mechanical agitation requires a substantial energy input, can be problematic with viscous cultures, has high operational and capital expenditure (OPEX/CAPEX), and causes hydrodynamic shear stress that can damage fungal mycelia [19]. Conversely, static liquid culture operates under unagitated conditions, and has notably lower energy inputs, equipment complexity, and capital costs [2,7]. However, under static conditions, fungal growth is restricted to the air–liquid interface due to the formation of a surface mycelial mat [19,21]. Here, nutrient and gas concentration gradients form in the liquid column. Upper aerial hyphae experience direct exposure to atmospheric oxygen, while the underlying submerged biomass can be severely oxygen-limited [19]. Consequently, while static systems offer an attractively simple and low-energy option for industrial wastewater valorisation, their inherent oxygen transfer constraints and potential for mass transfer resistance present distinct physiological trade-offs compared to conventional stirred tank bioreactors [7,19].

In addition to capital costs, costs of media components and sterilisation increase the OPEX of a bioprocess. Stillage residues represent an unutilised carbon source and culture medium for filamentous fungi, with the added advantage of sterility after distillation. Stillage is a dark-brown aqueous residue derived from ethanol production that has a low pH and high chemical oxygen demand (COD) and biological oxygen demand (BOD) [22]. While there is often ample complex carbon in stillage, nitrogen availability may be limited. Nitrogen supplementation may be necessary to achieve higher biomass and metabolite yields when using stillage as a culture medium [23,24].

This study evaluated the potential of Bundaberg rum distillery stillage as an alternative fermentation substrate to produce fungal biomass enriched with mycoprotein and L-carnitine using two GRAS fungal species: *Aspergillus oryzae* and *Rhizopus oligosporus*. A medium containing 25% stillage was supplemented with different nitrogen sources to identify the most effective strategy for maximising biomass yield, crude protein content, and L-carnitine production under both static and shake-flask culture. The optimal cultivation conditions were subsequently used to determine the time-course flux of fungal biomass, crude protein content, and L-carnitine content. Furthermore, the performance of stillage-based media was benchmarked against a complex synthetic medium (glucose, yeast extract, and malt extract). As there is limited knowledge on the performance of GRAS fungi in stillage, and none regarding static liquid culture, a comparative study of the impact of culture conditions on fungal yields helps to establish if stillage could serve as a cost-effective and sustainable substrate for producing fungal biomass, protein, and nutritional metabolites.

## 2. Materials and Methods

### 2.1. Chemicals

D-glucose, yeast extract, peptone, potassium phosphate monobasic, ammonium sulphate, magnesium sulphate, malt extract (Sigma-Aldrich, St. Louis, MO, USA), L-carnitine (Bulk supplement.com, Henderson, NV, USA), Tween 80, UPLC-grade hexane, acetonitrile (Thermo Fisher Scientific, Waltham, MA, USA).

### 2.2. Fungal Strains

GRAS filamentous fungal strains were isolated from two commercial products. *Aspergillus oryzae* was isolated from koji (Rice Culture, Nerang, QLD, Australia), while *Rhizopus oligosporus* was isolated from a tempeh starter culture (MRTempeh, Margaret River, WA, Australia). Fungal strains were cultured in Erlenmeyer flasks containing GYM media: glucose (10 g/L), yeast extract (5 g/L), malt extract (5 g/L), ammonium sulphate (0.5 g/L) and magnesium sulphate (0.5 g/L). Both strains were grown on plates containing GYM Agar, incubated at 28 °C for 7 days to produce spores. Spore suspensions were prepared by harvesting spores in a 20% glycerol solution containing 0.2% (*v*/*v*) Tween 80. The spore suspension was stored at −20 °C until use.

Inocula for the experiments were prepared by inoculating 1 mL from each spore suspension with 1 × 10^5^ spores/mL into a 250 mL volumetric flask containing 100 mL of preculture medium (GYM media containing 5% rum stillage), and they were incubated in a rotary shaker (150 rpm) at 28 °C for 12 h [20]. This culture was used as the inoculum for the experiment.

### 2.3. Shake-Flask Cultures

Media composed of 25% stillage (25% Raw Stillage) was supplemented with different nitrogen sources (yeast extract, peptone, urea or ammonium sulphate) based on stoichiometric nitrogen equivalents of yeast extract at 2 g/L. Unsupplemented 25% stillage was used as the baseline medium. Media (80 mL) was added to 250 mL Erlenmeyer flasks, autoclaved (30 min at 121 °C), aseptically inoculated, and placed in a shaker-incubator (Ratek OM25, Ratek Instruments, Melbourne, Australia) at 150 rpm at 28 °C [24]. The media was inoculated with 4 mL of actively-growing mycelia of *Aspergillus oryzae* or *Rhizopus oligosporus* that was washed using 25% raw stillage. Entire flasks were harvested after 48 or 96 h, washed with distilled water and freeze-dried (VirTis Bench Top Pro, Gardiner, NY, USA) at 50 µbar and −80 °C. Dried biomass was stored at −20 °C until L-carnitine extraction. All data are based on biological triplicates.

### 2.4. Static Plate Liquid Fermentation

Static plate cultures were conducted in square polystyrene petri plates (120 × 120 × 15 mm) containing 30 mL sterilised liquid media (25% Raw Stillage ± nitrogen supplements, as in Section 2.3), and had a 2.1 mm film depth. Media were inoculated with 1.5 mL of washed, actively growing mycelia produced as described in Section 2.3. Plates were incubated at 28 °C in a static laboratory incubator (Thermo Scientific Heratherm IGS 100, Thermo Fisher Scientific, Waltham, MA, USA). Fungal biomass was harvested from plates at 48 and 96 h, washed with distilled water, freeze-dried, and stored at −20 °C until L-carnitine extraction. All sample points were conducted as biological triplicates.

### 2.5. Time-Course Analysis of L-Carnitine Production

To assess the growth and the effect of L-carnitine synthesis with time, *Aspergillus oryzae* and *Rhizopus oligosporus* were cultured under both static and shake-flask conditions in three different media:

- 25% stillage (25% raw stillage);
- 25% stillage supplemented with yeast extract (2 g/L);
- a benchmark growth medium containing glucose (10 g/L), yeast extract (5 g/L) and malt extract (5 g/L) (GYM).

Cultures were harvested at five time intervals (24, 48, 72, 96 and 120 h). All the other parameters were kept constant as per the previous experiment (Section 2.3 and Section 2.4), and the harvested biomass was washed with distilled water and stored at −20 °C after freeze-drying.

### 2.6. L-Carnitine Extraction

Freeze-dried samples (30 mg) were ground into fine powder in 2 mL Eppendorf tubes by adding 3 mm tungsten carbide beads and shaking the samples at 30 Hz for 3 min in a Molecular Genomics Qiagen Tissue Lyser II (QIAGEN GmbH, Hilden, Germany). L-carnitine was extracted by adding 600 µL of water and sonicating the samples for 30 min at 60 °C in an Elmasonic sonicating water bath (Elma Schmidbauer GmbH, Singen, Germany). Lipids were removed in a biphasic extraction by adding 300 µL of hexane and shaking for 15 min at 150 rpm. The samples were centrifuged (13,000 rpm, 5 min), and the aqueous phase was collected after removing the hexane layer. Extracts were diluted using 80% acetonitrile containing 0.3% NH_4_OH (the running buffer of the UHPLC) and immediately processed in the UHPLC.

### 2.7. L-Carnitine Quantitation

The extracts were analysed using an ACQUITY UHPLC (Waters Corporation, Milford, MA, USA) system equipped with a PDA and ESI-MS detector. Chromatographic separation was achieved using the HILIC-Z column (2.0 mm × 150 mm, Agilent Technologies, Santa Clara, CA, USA) at 35 °C. The mobile phase consisted of 80% acetonitrile with 0.3% NH_4_OH at a flow rate of 0.4 mL/min for 15 min. Samples were maintained at 5 °C in the autosampler and 2 µL samples were injected. The PDA/UV spectra were recorded from 190 to 400 nm, and the quantitation wavelength used was 214 nm. The QDa mass detector was operated over the range of 85–300 amu in positive ion mode using selected ion recording (SIR) at 15 V cone voltage, with 162 at 15 V cone voltage to measure the chromatographic peaks. Calibration standards for L-carnitine (0.0025, 0.025, 0.05, 0.1, 0.25, 0.5, and 1 mg/mL) were prepared in Milli-Q water (Appendix B).

### 2.8. Crude Protein Quantification

Total nitrogen was determined using 3 mg of freeze-dried biomass samples processed in a FlashSMART Elemental Analyser (Thermo Scientific, MA, USA). The crude protein content was estimated by multiplying the total nitrogen by a nitrogen-to-protein factor of 6.25 [25].

### 2.9. Statistical Analysis

The data were analysed using GraphPad Prism (v10.4.1) and expressed as mean ± standard deviation. All the experiments were conducted in triplicate (*n* = 3), and the significant effect of nitrogen supplementation on the 25% stillage media was determined with two-way ANOVA. When significant effects were observed (*p* < 0.05), a multiple comparison test was used for *post hoc* analysis with the Bonferroni correction (α = 0.05).

## 3. Results

### 3.1. Stillage Concentration and Impact of Nitrogen Supplementation and Culture Conditions

In this study, Bundaberg rum stillage was evaluated as a carbon-rich substrate to produce fungal biomass. Two mould fungi (*Aspergillus oryzae* and *Rhizopus oligosporus*) were screened across a range of stillage concentrations to determine the highest concentration that did not inhibit growth. As growth inhibition was observed at stillage concentrations above 30 to 50%, 25% (*v*/*v*) stillage was selected as the working concentration for subsequent experiments (Appendix A). Growth inhibition may have been caused by inhibitory organic compounds such as polyphenols, melanoidins, or inorganic salts (sulphates and chlorides), which are known to interfere with biological activity [26].

**Figure 1:**
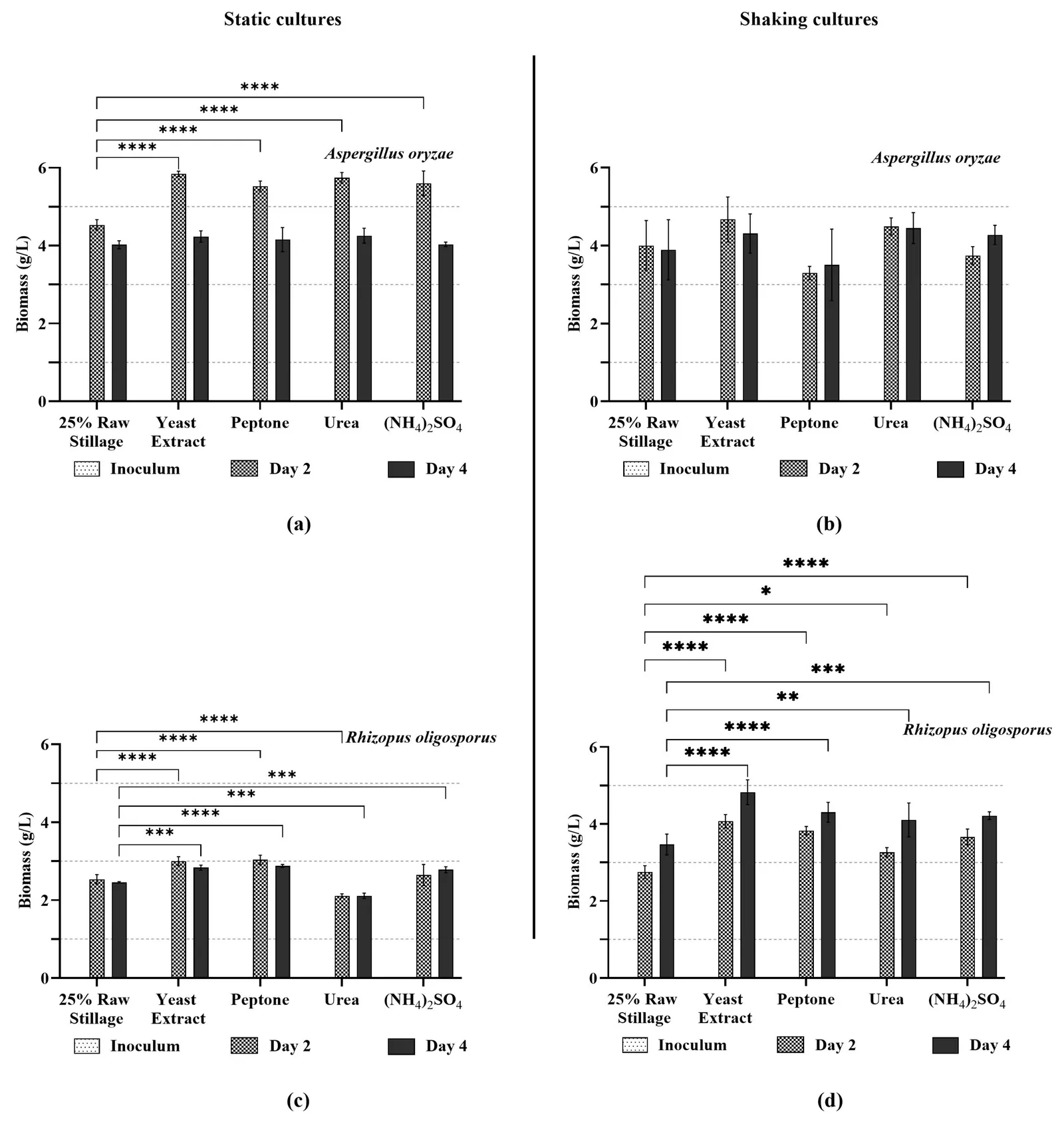
Fungal biomass production in 25% rum stillage with various nitrogen supplements by *Aspergillus oryzae* in static-liquid (**a**) and shake-flask cultures (**b**), and *Rhizopus oligosporus* in static-liquid (**c**) and shake-flask cultures (**d**) (*n* = 3). 25% Raw stillage was the control. Inoculum dry mass values at Time 0 not visible at the scale presented (*Aspergillus oryzae*: 0.028 g L^−1^; *Rhizopus oligosporus*: 0.017 g L^−1^). Statistical differences between treatments for each sample were determined using two-way ANOVA followed by Bonferroni correction for multiple comparisons (* *p <* 0.05, ** *p <* 0.1, *** *p <* 0.01, **** p < 0.001).

The impact of a nitrogen supplement on fungal growth was evaluated by adding either yeast extract, peptone, ammonium or urea to the stillage. The nitrogen supplement was based on a stoichiometric equivalent of 2 g/L yeast extract. In general, *A. oryzae* demonstrated a higher biomass yield of 5.84 ± 0.06 g/L in static cultures compared with a shake-flask culture, whereas *R. oligosporus* produced the highest biomass (4.82 ± 0.32 g/L) under shaking conditions (Figure 1).

For *Aspergillus oryzae* in supplemented stillage, greater biomass yields were generally observed after two days, whereas *Rhizopus oligosporus* biomass maximums were observed after four days. In unsupplemented stillage, the trends were similar, with minor changes in biomass for both strains under static and shake-flask cultures. Supplementing with yeast extract proved most advantageous to biomass yield for both strains under both fermentation conditions, with an increase of approximately 29% in *A. oryzae* and 19–39% in *R. oligosporus* compared with the non-supplemented control.

### 3.2. Impact of Nitrogen Supplement and Culture Conditions on L-Carnitine Synthesis

**Figure 2:**
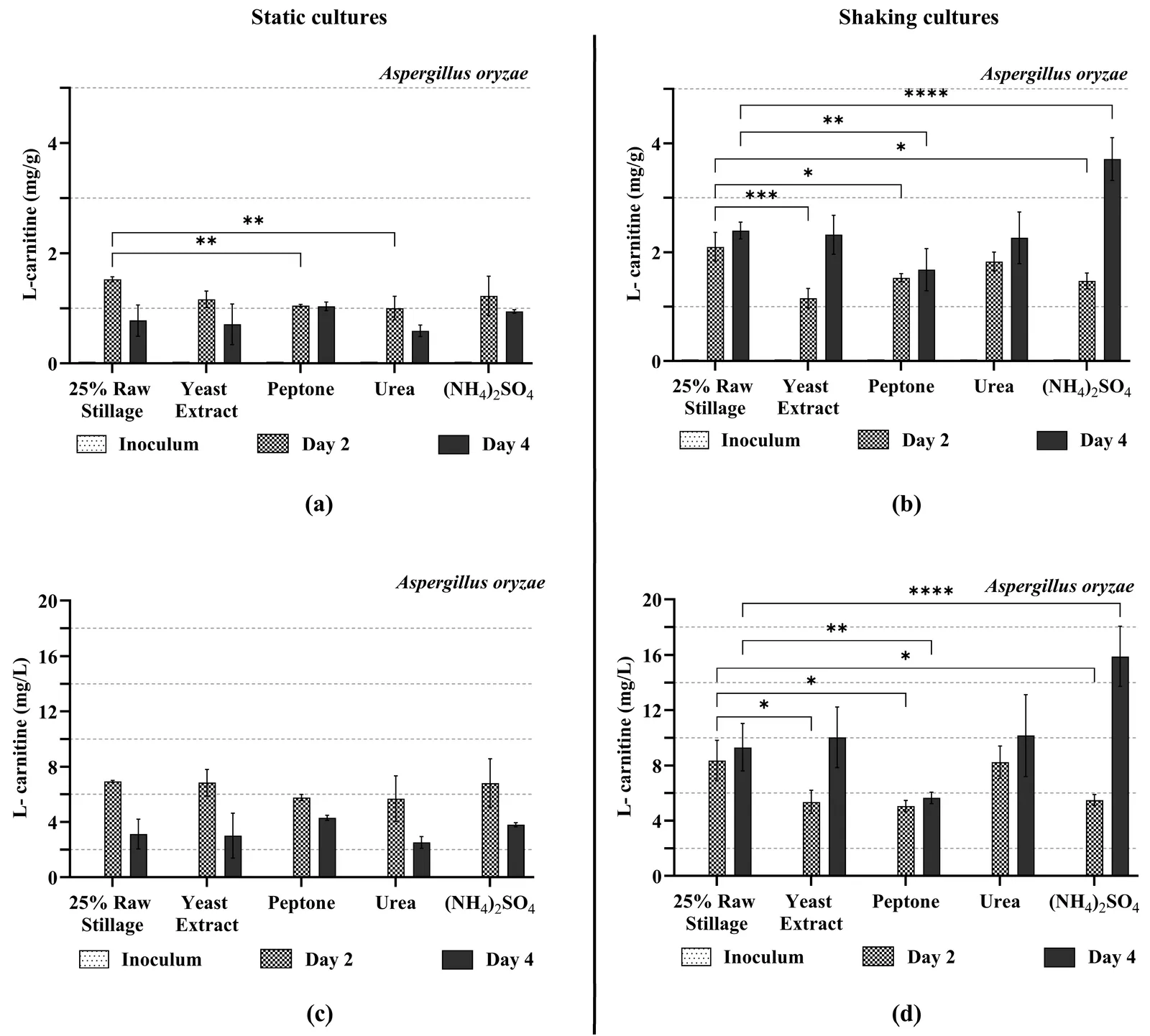
Comparative L-carnitine production of *Aspergillus oryzae* in 25% rum stillage supplemented with various nitrogen sources under static and shake-flask culture conditions over 4 days. L-carnitine yields in static liquid cultures (**a**), shake-flask cultures (**b**), and volumetric L-carnitine production in static liquid cultures (**c**) and shake-flask cultures (**d**) (*n* = 3). 25% Raw stillage was the control. Inoculum L-carnitine values at Time 0 for (**a**,**b**) (0.032 mg g^−1^ dry mass) and (**c**,**d**) (0.01 mg L^−1^) are not visible due to scale. Statistical differences between treatments for each sample were determined using two-way ANOVA followed by Bonferroni correction for multiple comparisons (* *p* < 0.05, ** *p* < 0.1, *** *p* < 0.01, **** *p* < 0.001).

The production of L-carnitine in *Aspergillus oryzae* and *Rhizopus oligosporus* was measured in static and shake-flask cultures grown in 25% (*v*/*v*) stillage, with or without a nitrogen supplement (yeast extract, peptone, ammonium or urea). In this initial screen, L-carnitine content was measured after two and four days. Under static culture, *R. oligosporus* had the highest L-carnitine content in media supplemented with ammonium (2.26 ± 0.15 mg/g dry biomass, Figure 2a), followed by peptone (2.15 ± 0.32 mg/g). In contrast, *A. oryzae* exhibited comparatively lower L-carnitine levels in static culture, with a maximum of 1.52 ± 0.04 mg/g in the unsupplemented stillage and 1.22 ± 0.35 mg/g in the ammonium-supplemented stillage (Figure 2a).

**Figure 3:**
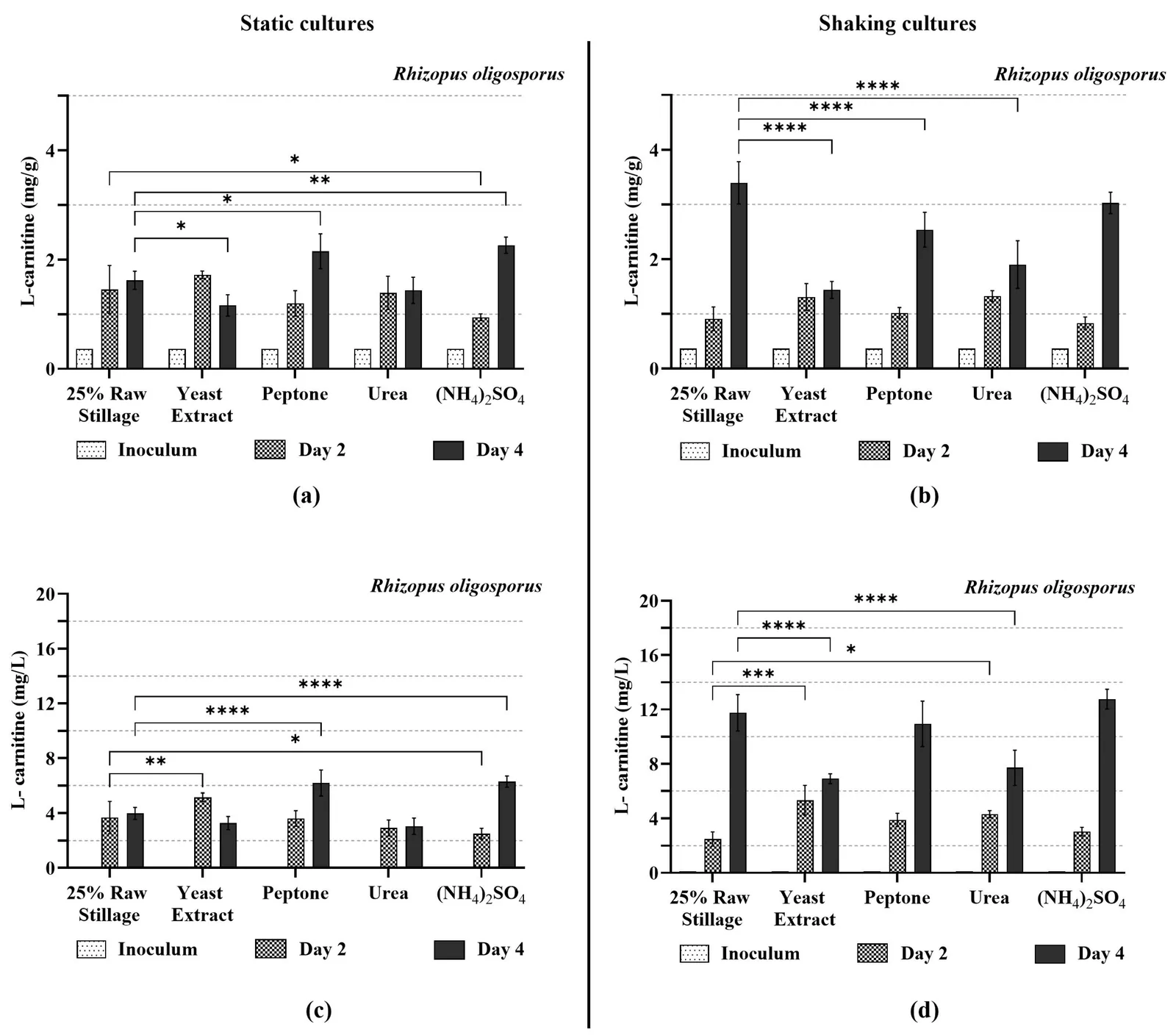
Comparative L-carnitine production by *Rhizopus oligosporus in* 25% rum stillage supplemented with various nitrogen sources under static and shake-flask cultures over four days. L-carnitine yields in static liquid cultures (**a**) and shake-flask cultures (**b**), and volumetric L-carnitine production in static liquid cultures (**c**) and shake-flask cultures (**d**), where *n* = 3. 25% Raw stillage was the control. Inoculum values at Time 0 for (**c**,**d**) (0.120 mg L^−1^) not visible due to scale. Statistical differences between treatments for each sample were determined using two-way ANOVA followed by Bonferroni correction for multiple comparisons (* *p* < 0.05, ** *p* < 0.1, *** *p* < 0.01, **** *p* < 0.001).

L-carnitine production improved with shake-flask cultures. *Aspergillus oryzae* achieved its highest L-carnitine content (3.71 ± 0.39 mg/g) with an ammonium supplement after four days (Figure 2b), followed by the unsupplemented control (2.39 ± 0.15 mg/g). *Rhizopus oligosporus* obtained its highest L-carnitine content (3.39 ± 0.38 mg/g) after four days in the unsupplemented stillage control medium (Figure 3b). With respect to volumetric yield of L-carnitine production, the highest yields were observed on the fourth day for both strains in the ammonium-supplemented medium for shake-flask cultures (Figure 2d and Figure 3d).

### 3.3. Effect of Nitrogen Supplement and Culture Conditions on Crude Protein Content

**Figure 4:**
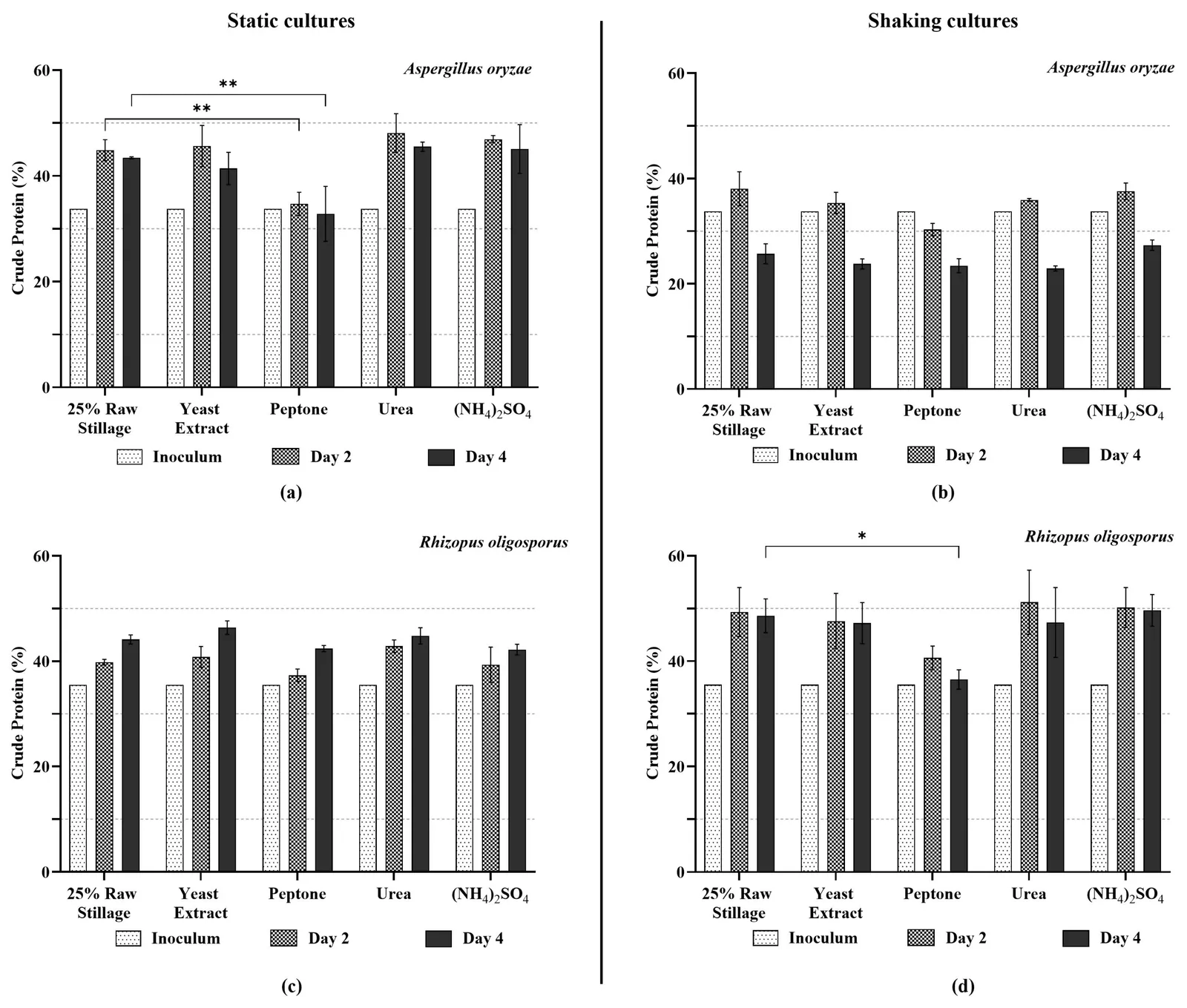
Crude protein content in *Aspergillus oryzae* biomass for static liquid cultures (**a**) and shake-flask cultures (**c**), and crude protein content in *Rhizopus oligosporus* biomass for static cultures (**b**) and shake-flask cultures (**d**), where *n* = 3. Statistical differences between treatments for each sample were determined using two-way ANOVA followed by Bonferroni correction for multiple comparisons (* *p* < 0.05, ** *p* < 0.1).

Previous studies have demonstrated that additional nitrogen can increase the crude protein content in mycelia [27]. In line with these findings, the present study examined the influence of nitrogen supplement, cultivation conditions, and incubation time on the crude protein content of the fungal strains. *Aspergillus oryzae* exhibited a higher crude protein content under static fermentation conditions, whereas *Rhizopus oligosporus* biomass had a higher protein content under shaking conditions (Figure 4). Most nitrogen supplements yielded a similar crude protein content in *A. oryzae*, with the highest value recorded at 48.1 ± 3.6%, while *R. oligosporus* achieved a maximum crude protein content of 51.2 ± 6.1%. In general, the culture method had the greatest impact on crude protein content. *Aspergillus oryzae* had a higher protein content with the static culture, while *Rhizopus oligosporus* had a slightly higher protein content with the shake-flask culture. In contrast, the nitrogen type had a limited impact, with crude protein values generally similar for all nitrogen sources as well as the control. Interestingly, a slight decrease was observed for peptone-supplemented cultures under static culture for *A. oryzae* and shake-flask culture for *R. oligosporus*.

### 3.4. Effect of Cultivation Time and Medium Composition on Biomass, Crude Protein, and L-Carnitine Production

The initial screening revealed distinct variations in biomass yield, L-carnitine concentration, and crude protein content between the mid-point (day 2) and the final sampling point (day 4) for both *A. oryzae* and *R. oligosporus*. Shake-flask and static liquid cultures were monitored over a five-day period to gain a deeper understanding of the relationship between biomass accumulation and L-carnitine synthesis and its potential degradation. Three media were evaluated, as follows:

- A benchmark control medium containing glucose, yeast extract and malt extract (GYM);
- 25% (*v*/*v*) stillage (25% Raw Stillage);
- 25% (*v*/*v*) stillage supplemented with 2 g/L yeast extract.

**Figure 5:**
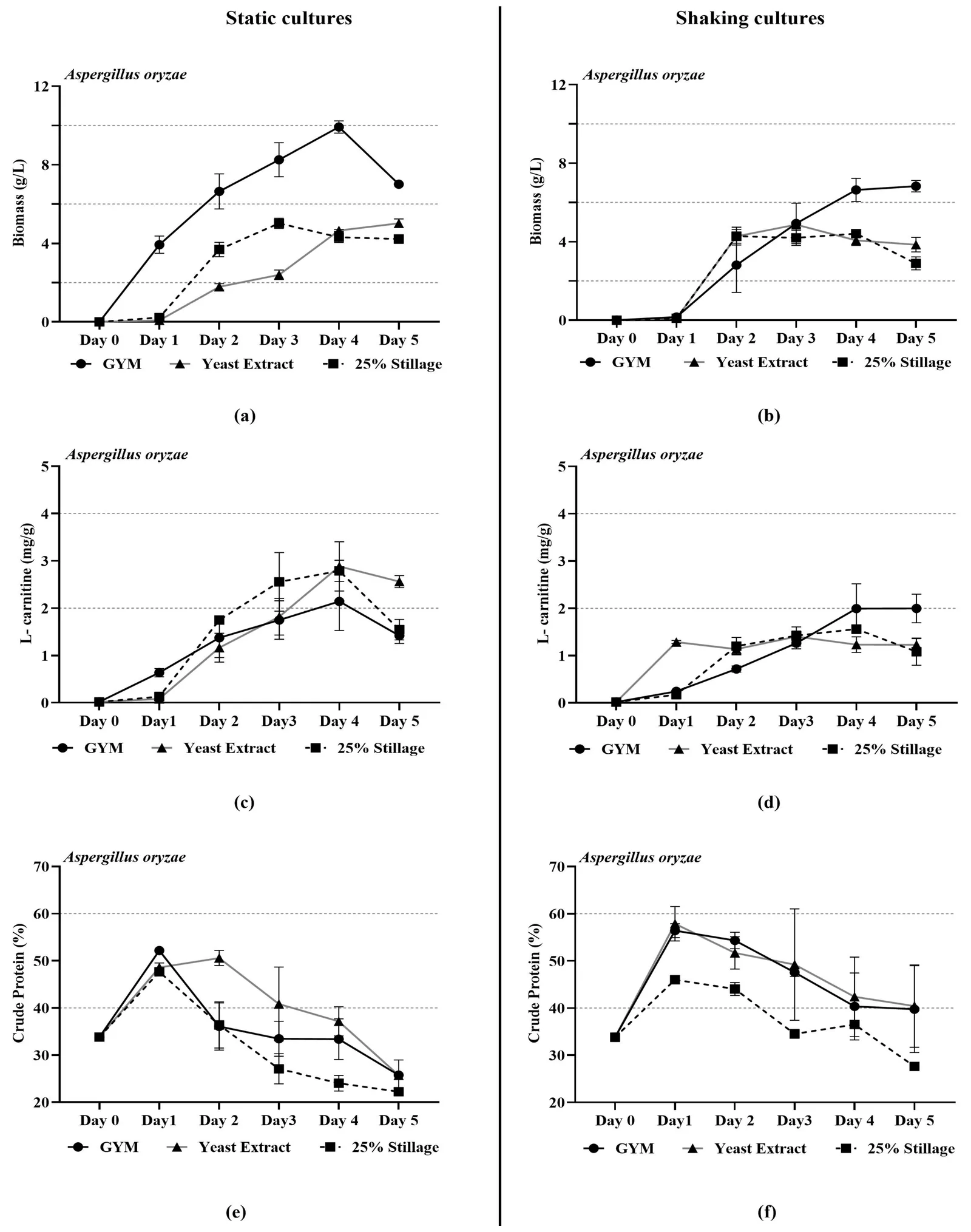
Time-course values for biomass, L-carnitine and crude protein yields for *Aspergillus oryzae* in static cultures (**a**,**c**,**e**) or shake-flask cultures (**b**,**d**,**f**), where *n* = 3.

**Figure 6:**
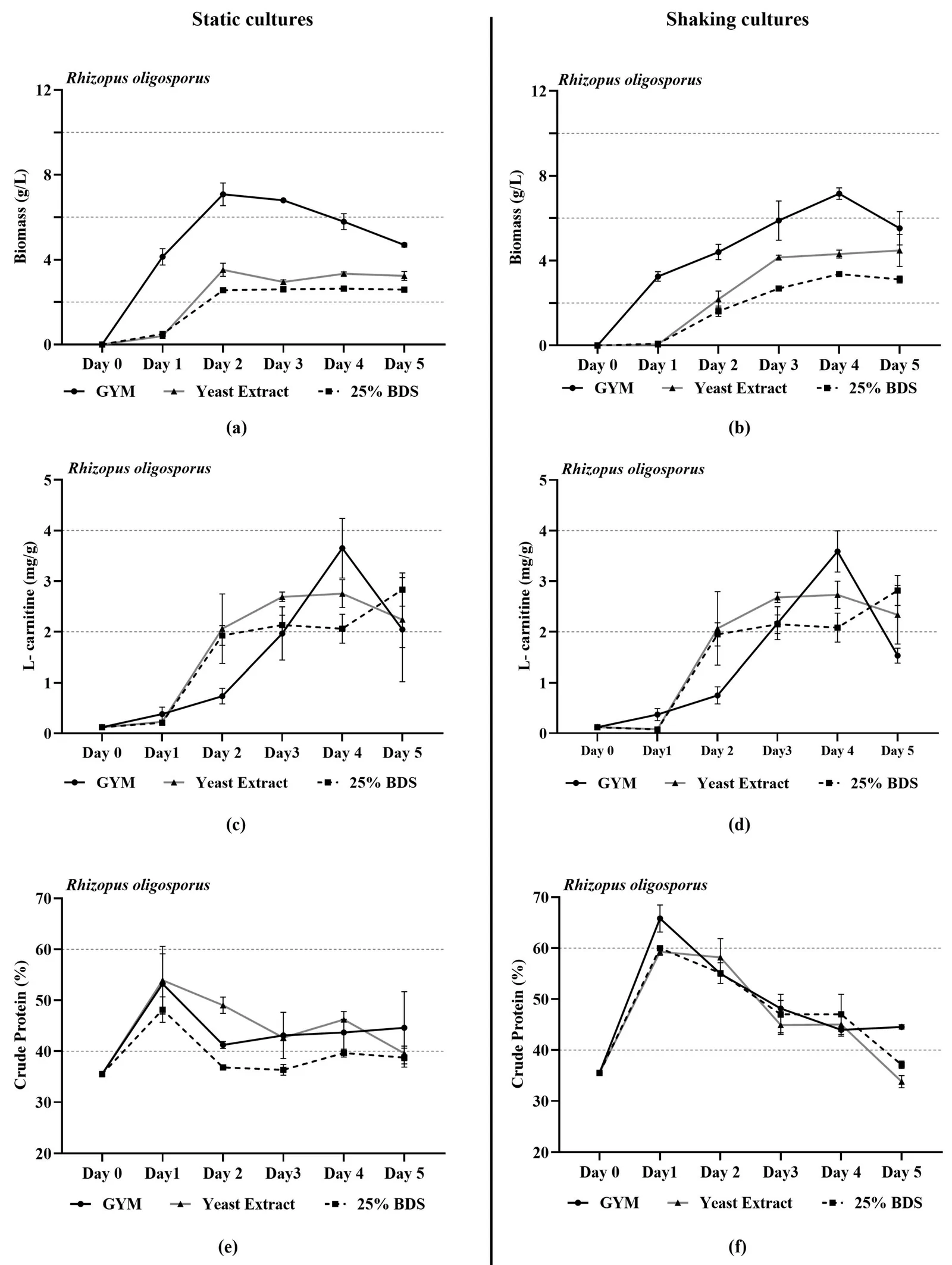
Time-course values for biomass, L-carnitine and crude protein yields for *Rhizopus oligosporus* in static cultures (**a**,**c**,**e**) and shake-flask cultures (**b**,**d**,**f**), where *n* = 3.

Biomass and L-carnitine content gradually increased for both shake-flask and static cultures for both species, reaching their respective maxima on day 4 before declining sharply by day 5 (Figure 5a–d and Figure 6a–d). The nutrient-rich GYM medium supported higher biomass production throughout the experimental period. In *A. oryzae*, the greatest L-carnitine content was obtained in static cultures using unsupplemented stillage. For *R. oligosporus,* a similar trend was observed for both static and shake-flask conditions, with a maximum L-carnitine yield on day 4.

The maxima for crude protein content were observed within the first 24 h for both strains, after which the protein content gradually declined. For *A. oryzae*, the highest crude protein content (57.9%) was measured for the shake-flask culture using stillage supplemented with yeast extract. This was slightly higher than that observed in the GYM medium. *Rhizopus oligosporus* crude protein content peaked at 24 h, with an average of 65.8% in GYM medium, 60.0% in 25% raw stillage and 59.3% in the yeast extract-supplemented stillage for shake-flask cultures. The crude protein content decrease was attributable to lower nutrient availability over the extended culture period.

## 4. Discussion

Carbon and nitrogen availability in culture media play a crucial role in fungal biomass and secondary metabolite production. This study used rum stillage resulting from distillation after molasses fermentation. The simple sugars from molasses are consumed during fermentation and ethanol production. The carbon remaining after distillation is more complex and less accessible than simple reducing sugars. Filamentous fungi are known for their ability to secrete various enzymes that degrade polymers and recalcitrant compounds into molecules that can be used for growth or energy [28]. Previous studies reported that stillage was deficient in nitrogen [29] and supplementing with inorganic nitrogen (ammonium or urea) enhanced biomass yields. Complex nitrogen supplements (e.g., yeast extract) supply additional nutrients, such as carbohydrates, amino acids, vitamins and trace elements, which collectively support more rapid and sustained fungal growth [30].

In this study, biomass yields obtained using rum stillage were consistent with previous studies using different carbon sources, fungal strains and culture conditions (Table 1). Compared to an earlier study with acetic acid as the sole carbon source, the biomass yields achieved in the present work were higher [31], while the current results were similar to yields of *A. oryzae* cultivated in sugarcane stillage (7.0 g/L over 10 days) [24]. The biomass yields of *R. oligosporus* in the current study were slightly lower than those of *A. oryzae*, but displayed a similar trend to that observed for *Rhizopus arrhizus* in previous research [19]. Collectively, these findings show that filamentous fungi possess considerable metabolic flexibility, and are capable of using industrial waste streams such as rum stillage for biomass and metabolite production.

A central finding of this investigation relates to the physiological divergence between *A. oryzae* and *R. oligosporus* when grown in a static-liquid or shake-flask culture. *Aspergillus oryzae* accumulated significantly greater biomass with a dense, interconnected hyphal mat under static conditions (5.84 g/L in yeast extract-supplemented stillage) than in shake-flasks (4.30 g/L). This may be due to the ability of *Aspergillus* species to develop cohesive surface-associated mycelial networks during static cultivation. In several *Aspergillus* species, these surface-associated structures have also been linked to the production of an extracellular matrix containing polysaccharides (including galactomannan and glucans), hydrophobins, and other extracellular components that facilitate hyphal adhesion and biofilm development [21,32]. These characteristics likely contributed to the greater biomass production of *A. oryzae* under static culture in the present study. The aerial mat allows for improved access to atmospheric oxygen at the gas–liquid interface, bypassing the rate-limiting dissolved oxygen constraints of the liquid phase, while still being able to absorb nutrients from the stillage [30]. Furthermore, static conditions avoid mechanical shear stress, which allows enzyme secretion and uninterrupted hyphal elongation of the mycelial network [32,33]. In contrast, *Rhizopus oligosporus* exhibited a clear preference for agitated shake-flask culture, achieving a maximum biomass yield of 4.82 g/L (compared to 3.03 g/L in static culture). This is possibly due to the dependence of *R. oligosporus* on oxygen transfer and nutrient mixing during submerged culture, which directly impacts fungal morphology and metabolic activity [34,35].

The use of static and agitated culture also impacted secondary metabolism, specifically L-carnitine biosynthesis. Although *A. oryzae* produced more biomass in the static culture, its maximum L-carnitine content of 3.71 mg/g biomass (15.8 mg/L volumetric yield) under shake-flask culture with an ammonium supplement. In static culture, *A. oryzae* only produced 1.22 mg/g with the same nitrogen supplement. L-carnitine synthesis requires trimethyl-lysine dioxygenase and γ-butyro-betaine dioxygenase, both of which are oxygen-dependent enzymes that require molecular oxygen as a co-substrate [7,9,11]. While the surface hyphae in static mats encounter adequate atmospheric oxygen, the sub-surface mycelium would have been oxygen-limited and functioning under microaerophilic conditions. This would restrict flux through oxygen-dependent biosynthetic pathways [7,29]. Fluid agitation in shake-flasks improves dissolved oxygen availability in the bulk liquid, improving the function of L-carnitine hydroxylases [7,8]. For *R. oligosporus*, the maximum L-carnitine synthesis of 3.39 mg/g biomass (12.7 mg/L volumetric yield) was achieved using shake-flask cultures in unsupplemented stillage. This indicates that robust bulk oxygenation is required to improve L-carnitine yields per unit biomass for both the Ascomycete and Zygomycete species used in this study, even when static conditions favoured vegetative biomass accumulation in species like *A. oryzae* [7,15,29].

Generally, higher crude protein contents are obtained with N-supplemented media. Complex nitrogen sources such as peptone and yeast extract contain amino acids, vitamins, carbohydrates, and trace elements that can stimulate intracellular protein synthesis [36]. Consistent with previous research, their inclusion in media can significantly increase the crude protein content of fungal biomass, with reported improvements of up to 1.6 times compared to unsupplemented controls [37]. Inorganic nitrogen sources such as urea and ammonium can provide a more specific carbon-to-nitrogen (C:N) ratio, which can support fungal biomass and protein synthesis. Crude protein analysis is an important parameter for assessing the nutritional value of fungal biomass, particularly when it is intended for use as a protein source [20]. The crude protein content in the current study was estimated from the total nitrogen content. Unlike extraction-based protein assays, elemental nitrogen analysis does not require protein solubilisation and is less influenced by extraction efficiency. However, as total nitrogen (rather than protein-specific nitrogen) is measured, the estimated crude protein may be slightly over-estimated. Nonetheless, this remains a widely accepted and standard approach [38].

Both fungal species exhibited similar peaks in crude protein content within the initial 24 h (57.9% in *A. oryzae* and 60.0% in *R. oligosporus* in stillage). This was followed by a progressive decline over time (Figure 5e,f and Figure 6e,f). Biomass from supplemented and unsupplemented raw stillage had lower crude protein contents than the GYM medium, potentially due to the limited available nitrogen or the C:N ratio in the culture medium [36,37,39]. Culture conditions impacted crude protein content. In *A. oryzae*, the static liquid culture supported slightly higher crude protein levels during the mid-exponential phase, while the shake-flask culture supported the higher protein content in *R. oryzae*. The temporal variation in crude protein content did not directly correlate with biomass accumulation. The higher protein content observed after 24 h was likely due to a greater proportion of nitrogen-rich cellular components in the newly formed biomass. As growth progressed, the accumulation of structural materials diluted the relative protein content, resulting in a lower protein content. Therefore, the decline in protein content represents a compositional shift in the biomass. The rapid initial protein accumulation reflects energy investment in ribosome and primary metabolic enzyme synthesis during early growth and hyphal extension [35,36]. The rapid initial protein accumulation is consistent with the active hyphal growth phase, during which the proteins and enzymes involved in structural synthesis are continuously delivered to the growing hyphal tip to sustain biomass formation [40]. As nutrients become depleted and biomass accumulates, intracellular protein is re-mobilised or diluted by storage carbohydrates and cell wall chitin [41]. Agitation enhances shear-induced cell wall thickening and turnover in *R. oligosporus*, whereas mat formation with a static culture of *A. oryzae* promotes a stable protein-rich structural matrix [29,37]. From an industrial bioprocessing perspective, these results indicate that harvesting biomass at early growth stages (24 to 48 h) maximises mycoprotein nutritional density, whereas optimising L-carnitine accumulation requires extended fermentation (up to 96 h) under agitated, well-oxygenated conditions [7,37,38].

Previous studies have reported a significant decline in protein productivity with increasing cultivation time. The highest productivity was achieved within the shortest cultivation period (four days) for *Aspergillus* species grown in sugarcane vinasse [20]. Similarly, for *A. oryzae* cultured in spent sulphite liquor, a higher crude protein content was observed when the fungal biomass yield was lower. These findings corroborate higher protein synthesis and accumulation in younger mycelial biomass [42]. However, earlier studies showed that the crude protein content in *Rhizopus* species remained relatively stable (49% to 55%) throughout the cultivation period when using a starch-based thin stillage. In separate studies, aeration rate did not have a significant effect on crude protein synthesis when vinasse was used as the substrate [35,43]. While crude protein percentage tends to, at best, remain stable or decrease over an extended cultivation period, protein productivity generally declines significantly with longer fermentation time. From this perspective, the most economically efficient production period for protein harvest typically occurs within a shorter duration (from 14 to 72 h), depending on the fungal strain and the substrate used [20,36,39].

Nitrogen regulates several aspects of fungal metabolism, including enzyme production and secondary metabolite biosynthesis. Previous studies reported that substrate composition significantly affected L-carnitine synthesis in filamentous fungi such as *Neurospora crassa*, *Aspergillus oryzae*, *Rhizopus oligosporus*, and *Neurospora intermedia* [9,16,17,44,45]. In submerged culture, supplementing with yeast extract, methionine, or lysine improved L-carnitine yields in *A. oryzae*, while *R. oligosporus* and *N. intermedia* exhibited a comparatively weaker response [7]. *Aspergillus oryzae* can metabolise inorganic nitrogen compounds (ammonium and nitrate), complex nitrogen sources and most L-amino acids, which support rapid conidia germination and extensive hyphal development [31,46]. This may explain the rapid early growth of *A. oryzae* in media containing stillage, and slower biomass accumulation at later stages. In contrast, the extended biomass accumulation observed in GYM medium likely reflects the continued availability of carbon, nitrogen and micronutrients, which provided nutrients later into the cultivation period and supported sustained fungal growth. Residual carbon and nitrogen concentrations and fungal viability were not measured in this study. As such, the specific mechanism responsible for the decline in biomass during the later stages of cultivation cannot be conclusively stated.

The L-carnitine yields obtained in the current study using *A. oryzae* (3.71 mg/g) and *R. oligosporus* (3.39 mg/g) with 25% rum stillage in shake-flask cultures were slightly higher than those previously reported. Rousta et al. [7] reported a maximum L-carnitine production of 3.0 mg/g using *A. oryzae* in a semi-synthetic medium under submerged conditions, and only 1.3 mg/g with a strain of *R. oligosporus*. A previously published study of solid-state fermentation of ginseng using *R. oligosporus* only detected L-carnitine after fungal fermentation. Similarly, higher L-carnitine content was observed with oyster mushrooms that were cultivated on buckwheat that was pre-fermented with *R. oligosporus*. In addition, quinoa fermented with *R. oligosporus* for three days was reported to yield the highest concentration of L-carnitine and GABA (Gamma aminobutyric acid), indicating that a relatively short time period can be optimal for metabolite production (Table 2) [16,17,18]. Collectively, these findings prove that the substrate fermented with *R. oligosporus* exhibits an enriched biochemical profile beyond the L-carnitine alone. Interestingly, in this study, *R. oligosporus* exhibited the highest yield of L-carnitine in 25% stillage without any additional nitrogen supplement, suggesting that the moderate nitrogen limitations in the stillage were sufficient for the synthesis of metabolites, including L-carnitine, in the biomass produced. These findings demonstrate the potential use of rum stillage as a cost-effective substrate for producing L-carnitine in filamentous fungi.

Static liquid and shake-flask culture are fundamentally different with respect to hydrodynamics and oxygen/nutrient mass transfer. These factors strongly affect filamentous fungal morphology, biomass accumulation and secondary metabolite production. Under a static culture, oxygen diffusion is restricted to the air–liquid interface, favouring the development of surface mycelial mats with minimal shear. This environment can benefit *Aspergillus oryzae*, which readily forms cohesive surface growth and efficiently exploits nutrient gradients. In contrast, agitation improves oxygen transfer, homogenises nutrient dispersion and minimises mass-transfer limitations, which improved the performance of *Rhizopus oligosporus* in shake-flask culture. The increased oxygen availability is also consistent with the higher L-carnitine titres observed under agitation, because aerobic metabolism supports precursor availability and energy generation for biosynthesis. These findings suggest that a cultivation strategy should be selected according to the desired product and strain preferences. A static culture is favoured for economical biomass production using strains where surface growth is advantageous, while the agitated submerged culture improved metabolite productivity via oxygen and nutrient mass transfer [5,33,47].

Cultivation time had a significant impact on the final L-carnitine yield in the biomass. L-carnitine production generally increased up to day four, where maximum biomass was achieved and the primary carbon sources were still available, after which it declined markedly [7]. Interestingly, both *A. oryzae* and *R. oligosporus* exhibited the highest L-carnitine concentration in 25% raw stillage media, rather than the nutrient-rich GYM, indicating that moderate nutrient limitation may stimulate the synthesis of L-carnitine or related metabolites. The decline observed beyond 96 h suggests that a shorter fermentation period would be advantageous for efficient L-carnitine production. It also clearly indicates that nutrient availability, culture mode and target product must all be considered when selecting a production process for fungal products.

**Table 1:** Media components and culture conditions for *Aspergillus* and *Rhizopus* species.

Strain	Primary Carbon	Culture Conditions	Time	Nitrogen Source	Dry Biomass Yield (g/L)	Reference
*A.oryzae*	25% Rum stillage	Static culture 28 °C	48 h	Yeast extract	5.87 ± 0.28	Current study
				Urea	5.61 ± 0.45	
				(NH_4_)_2_SO_4_	5.6 ± 0.31	
				Peptone	5.52 ± 0.13	
				-	4.54 ± 0.13	
		Shake-flask 150 rpm/28 °C	48 h	Urea	4.49 ± 0.21	
				-	4.0 ± 0.64	
			96 h	Yeast extract	4.30 ± 0.50	
				(NH_4_)_2_SO_4_	4.27 ± 0.24	
				Peptone	3.50 ± 0.92	
*R. oligosporus*	25% Rum stillage	Static culture 28 °C	48 h	Yeast extract	3.0 ± 0.11	
				Peptone	3.03 ± 0.12	
				-	2.53 ± 0.12	
			96 h	Urea	2.11 ± 0.05	
				(NH_4_)_2_SO_4_	2.77 ± 0.07	
		Shake-flask 150 rpm/28 °C	96 h	Yeast extract	4.82 ± 0.32	
				Peptone	4.30 ± 0.25	
				-	3.46 ± 0.26	
				Urea	4.10 ± 0.44	
				(NH_4_)_2_SO_4_	4.21 ± 0.10	
*A. oryzae*	45 g/L acetic acid	Shake-flask 100 rpm/30 °C	48 h	(NH_4_)_2_SO_4_	0.92 ± 0.13	[31]
				NaNO_3_	0.48 ± 0.06	
				Urea	0.9 ± 0.23	
				Yeast extract	3.59 ± 0.14	
				Peptone	3.58 ± 0.05	
*A. niger*	Sugar cane distillery wastewater/25–50%	Shake-flask 150 rpm/28 °C	Day 10	Yeast extract (2 g/L)	7	[24]
*A. niger*	Cassava whey Conc/29.2%	Orbital shaker 120 rpm/28 °C	Day 5	Yeast extract	2.75	[48]
				(NH_4_)_2_SO_4_	2	
			24 h	NaNO_3_	0.6	
*A. oryzae*	Olive oil mill wastewater/undiluted	Shake-flask 125 rpm/35 °C	96 h	-	8.1	[49]
*R. arrizuz*	Waste potato starch/90%	Shake-flask 150 rpm/30 °C	48 h	Yeast extract	4.9	[19]
				Peptone	4.6	
				Urea	2.7	
				(NH_4_)_2_SO_4_	2.75	
				NaNO_3_	2.25	
*R. delemar*	Olive oil mill wastewater/undiluted	Shake-flask 125 rpm/35 °C	96 h	-	0.49	[49]

**Table 2:** L-carnitine yields using filamentous fungi in previous studies.

Fungal Strain	Substrate	L-Carnitine Yield	Fermentation	Time	References
*A. oryzae*	25% raw stillage with (NH_4_)_2_SO_4_	3.71 mg/g ± 0.39 dry mass	Shake flask 150 rpm/28 °C	4 days	Current study
*R. oligosporus*	25% raw stillage	3.39 mg/g ± 0.38 dry mass			
*A. oryzae*	Semi-synthetic medium (30 g/L of glucose, 5 g/L yeast extract, 4.55 g/L NaNO_3_)	3 mg/g dry mass	Submerged	2 days	[7]
*R. oryzae*		1.3 mg/g dry mass			
*R. oligosporus*		1 mg/g dry mass			
*N. intermedia*		0.4 mg/g dry mass			
*R. oligosporus*	Wild ginseng	0.630 mg/g substrate	Solid-state	14 days	[17]
*R. oligosporus* *P. ostreatus*	Buckwheat	0.201 mg/g substrate	Solid state	38 days	[18]
*R. oligosporus*	Quinoa	0.315 mg/g substrate	Solid state	3 days	[16]

Conventional carbon sources (glucose and sucrose) and complex nitrogen sources (yeast extract and peptone) represent a substantial proportion of fermentation medium costs. In contrast, agro-industrial wastewaters often exhibit high chemical oxygen demand (COD), and naturally contain abundant organic carbon, nitrogenous compounds, and trace minerals, enabling the partial or complete replacement of purchased sugars and mineral supplements. Consequently, the use of such waste streams can significantly reduce medium costs. Techno-economic assessments of industrial submerged fermentation processes have shown that culture media typically account for 30–40% of total operating expenditures [50,51]. In conventional industrial and laboratory bioprocesses, liquid media and bioreactor assemblies are sterilised by moist heat treatment at 121 °C for approximately 20 min under pressure [52]. This sterilisation step incurs significant energy and operating costs. However, these costs may be largely avoided when using stillage, which can be considered effectively heat-sterilised due to high-temperature processing during ethanol recovery. As a general indicator, heating 1000 L of medium to 121 °C requires approximately 120 kWh of energy, while maintaining this temperature for 20 min requires an additional 6–23 kWh, corresponding to a total energy demand of approximately 145–200 kWh per batch. At an electricity cost of US$0.10 kWh^−1^, this equates to approximately US$15–20 per 1000 L batch when using a direct electric industrial boiler. Realising these savings would, however, require the fungal biomass production facility to be co-located or close to a distillery.

Beyond savings associated with medium formulation and sterilisation, the present research suggests that a thin-layer liquid cultivation system may provide a cost-effective platform for fungal biomass production. Compared to conventional agitated submerged cultures, which are typically scaled in continuously stirred-tank reactors, thin-layer systems offer potential reductions in both capital and operating expenditure. Replacing pressurised, mechanically sealed stirred vessels with modular shallow trays or troughs substantially lowers equipment and infrastructure costs. Operational energy demands are likewise reduced because thin liquid layers eliminate the need for the energy-intensive mixing of increasingly viscous fungal broths, relying instead on limited fluid circulation and passive headspace aeration. Furthermore, fungal growth occurs as a dense, cohesive surface mat rather than dispersed suspended biomass, thereby reducing downstream dewatering and drying requirements. Collectively, these factors suggest that thin-layer liquid cultivation may offer a more economically efficient alternative to conventional submerged fermentation for fungal biomass production, particularly when integrated with low-cost agro-industrial side stream. However, these systems still require rigorous validation for scale-up, and may better suit mid-sized distilleries or a slipstream volume from larger industrial plants.

## 5. Conclusions

This study demonstrated that Bundaberg rum stillage can be used as a low-cost substrate for biomass and L-carnitine production by *Aspergillus oryzae* and *Rhizopus oligosporus*. Both selected strains are classified as GRAS (Generally Recognised as Safe) species. They exhibited distinct physiological and metabolic responses to media types and culture conditions. Under static conditions, *A. oryzae* achieved superior biomass production, reflecting its capacity to thrive under oxygen constraints. In contrast, *R. oligosporus* displayed better growth under shake-flask conditions, consistent with better oxygen transfer in agitated systems. Maximum biomass production for both fungi was obtained in a benchmark synthetic complex medium (glucose, yeast extract and malt extract)., which highlights the significance of balanced nutrient availability for optimal fungal growth. Nevertheless, rum stillage produced competitive yields with minor ammonium or yeast extract supplements. Although the biomass yields derived from stillage were slightly lower than those from the synthetic medium, the reduced cost of the substrate highlights its potential for sustainable, large-scale cultivation.

L-carnitine is a naturally occurring, water-soluble derivative of amino acids [53] that is vital for energy production, transporting fatty acids, and reducing oxidative stress [8,18]. It is critical for maintaining metabolic health and is commonly used as a dietary supplement to enhance physical performance, cardiovascular health and weight management [9]. Both *A. oryzae* and *R. oligosporus* exhibited increased metabolic activity in shake-flask conditions, resulting in improved L-carnitine synthesis. A nitrogen supplement further promoted metabolite production; *A. oryzae* achieved its highest L-carnitine production in the stillage supplemented with ammonium, while *R. oligosporus* obtained its highest yield in an unsupplemented stillage, surpassing that obtained in the benchmark medium.

From a scale-up and techno-economic standpoint, submerged fermentation represents a well-studied and trialled production process. While it is expensive, submerged fermentation generally provides superior process reproducibility, scalability, and volumetric productivity. While solid-state and static systems face challenges in industrial deployment (non-uniform nutrient distribution, restricted oxygen transfer, limited process control), the low capital expenditure is very appealing. Current chemical synthesis routes for L-carnitine typically produce racemic mixtures containing D-carnitine and generate substantial quantities of chemical waste [14]. The bioprocess proposed in this study offers a more environmentally benign and selective alternative. The fungal biomass generated under all treatment conditions contained approximately 40–50% mycoprotein content, confirming its suitability for use as a high-protein microbial ingredient for feed applications [7]. Collectively, these findings demonstrate that rum stillage can be effectively valorised as a sustainable and low-cost substrate for *A. oryzae* and *R. oligosporus*, enabling the integrated production of L-carnitine and protein-rich fungal biomass. However, a techno-economic feasibility study is required to determine the scale best suited for static or agitated stillage-based fungal biomass production. Future research should focus on nutrient utilisation dynamics and fungal viability during cultivation to determine correlation with impacts on biomass and L-carnitine yields at later growth stages.

## Figures and Tables

**Figure A1:**
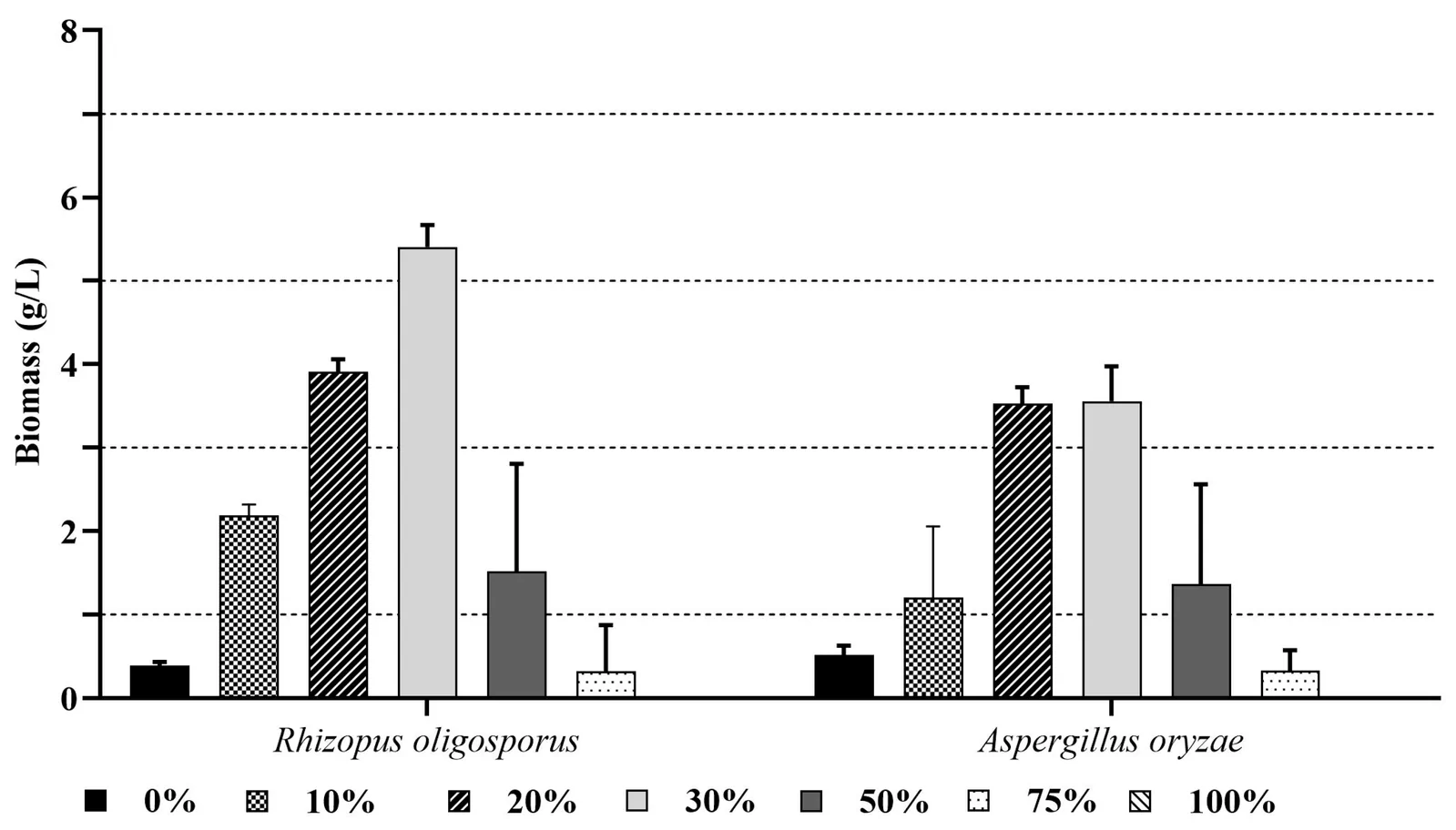
Preliminary assessment of biomass yields (g/L) of two filamentous fungi strains (*Rhizopus oligosporus* and *Aspergillus oryzae*) after 12 days of submerged cultivation in media with increasing concentrations of stillage (0–100%). Fungi were cultured in 24-well deep well plates at 25 °C while shaking at 150 rpm. Biomass was collected in a fine-meshed sieve, rinsed with distilled water and oven-dried (*n* = 3).

**Figure A2:**
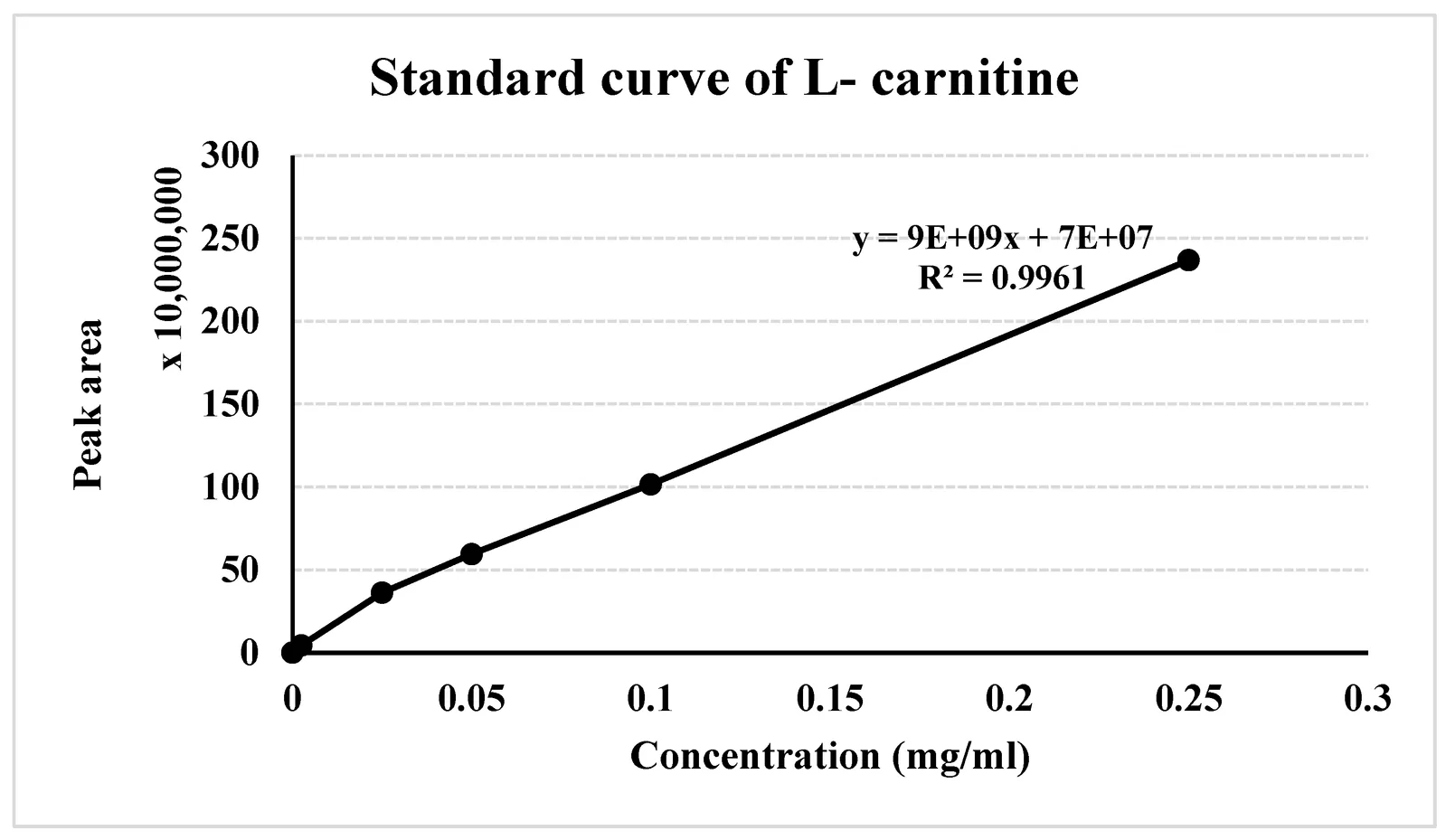
Standard curve of L-carnitine.

## Data Availability

The original data presented in the study are included in the article; further inquiries can be directed to the corresponding author.

## Abbreviations

The following abbreviations are used in this manuscript:

GRAS	Generally Recognised as Safe
GYM	Glucose, Yeast extract, Malt extract
UHPLC	Ultra-High Performance Liquid Chromatography
GABA	Gamma aminobutyric acid
